# Does urban greenness reduce loneliness and social isolation among Canadians? A cross-sectional study of middle-aged and older adults of the Canadian Longitudinal Study on Aging (CLSA)

**DOI:** 10.17269/s41997-023-00841-x

**Published:** 2023-12-29

**Authors:** Paul J. Villeneuve, Gagan K. Gill, Susanna A. Cottagiri, Robert Dales, Daniel Rainham, Nancy A. Ross, Habibe Dogan, Lauren E. Griffith, Parminder Raina, Dan L. Crouse

**Affiliations:** 1 Department of Neuroscience, Carleton University, Ottawa, ON Canada; 2 Department of Public Health Sciences, Queen’s University, Kingston, ON Canada; 3 Population Studies Division, Environmental Health Science & Research Bureau, Health Canada, Ottawa, ON Canada; 4 University of Ottawa and Ottawa Hospital Research Institute, Ottawa, ON Canada; 5 Faculty of Health, School of Health and Human Performance, Dalhousie University, Halifax, NS Canada; 6 Healthy Populations Institute, Dalhousie University, Halifax, NS Canada; 7 Department of Health Research Methods, Evidence, and Impact, McMaster University, Hamilton, ON Canada; 8 Labarge Centre for Mobility in Aging, McMaster University, Hamilton, ON Canada; 9 McMaster Institute for Research on Aging, McMaster University, Hamilton, ON Canada; 10 Health Effects Institute, Boston, MA USA

**Keywords:** Greenness, Loneliness, CLSA, Urban health, Verdure, solitude, ÉLCV, santé en milieu urbain

## Abstract

**Objectives:**

Urban greenness has been shown to confer many health benefits including reduced risks of chronic disease, depression, anxiety, and, in a limited number of studies, loneliness. In this first Canadian study on this topic, we investigated associations between residential surrounding greenness and loneliness and social isolation among older adults.

**Methods:**

This cross-sectional analysis of the Canadian Longitudinal Study on Aging included 26,811 urban participants between 45 and 86 years of age. The Normalized Difference Vegetation Index (NDVI), a measure of greenness, was assigned to participants’ residential addresses using a buffer distance of 500 m. We evaluated associations between the NDVI and (i) self-reported loneliness using the Center for Epidemiological Studies Depression Scale, (ii) whether participants reported “feeling lonely living in the local area”, and (iii) social isolation. Logistic regression models were used to characterize associations between greenness and loneliness/social isolation while adjusting for individual socio-economic and health behaviours.

**Results:**

Overall, 10.8% of participants perceived being lonely, while 6.5% reported “feeling lonely in their local area”. Furthermore, 16.2% of participants were characterized as being socially isolated. In adjusted models, we observed no statistically significant difference (odds ratio (OR) = 0.99; 95% confidence interval (CI) 0.93–1.04) in self-reported loneliness in relation to an interquartile range (IQR) increase of NDVI (0.06). However, for the same change in greenness, there was a 15% (OR = 0.85; 95% CI 0.72–0.99) reduced risk for participants who strongly agreed with “feeling lonely living in the local area”. For social isolation, for an IQR increase in the NDVI, we observed a 7% (OR = 0.93; 95% CI 0.88–0.97) reduction in prevalence.

**Conclusion:**

Our findings suggest that urban greenness plays a role in reducing loneliness and social isolation among Canadian urbanites.

**Supplementary Information:**

The online version contains supplementary material available at 10.17269/s41997-023-00841-x.

## Résumé

**Objectifs:**

Il est démontré que la verdure urbaine confère de nombreux avantages pour la santé; elle réduit notamment les risques de maladies chroniques, de dépression et d’anxiété et, selon un petit nombre d’études, le risque de solitude. Dans cette première étude canadienne sur le sujet, nous avons étudié les associations entre la verdure de l’environnement résidentiel et la solitude et l’isolement social chez les adultes d’âge mûr.

**Méthode:**

Cette analyse transversale de l’Étude longitudinale canadienne sur le vieillissement a inclus 26 811 participantes et participants urbains de 45 à 86 ans. L’indice de végétation par différence normalisée (IVDN), un indicateur de verdure, a été assigné à l’adresse domiciliaire dans une zone tampon de 500 m. Nous avons évalué les associations entre l’IVDN et i) la solitude autodéclarée selon l’échelle de dépression du Center for Epidemiological Studies, ii) le fait de déclarer « vivre de la solitude dans sa zone locale » et iii) l’isolement social. Des modèles de régression logistique ont servi à caractériser les associations entre la verdure et la solitude/l’isolement social, et nous avons apporté des ajustements pour tenir compte du statut socioéconomique et des comportements de santé individuels.

**Résultats:**

Globalement, 10,8 % des participantes et des participants se sentaient seuls, et 6,5 % disaient « vivre de la solitude dans leur zone locale ». De plus, 16,2 % des participantes et des participants ont été caractérisés comme étant socialement isolés. Dans nos modèles ajustés, nous n’avons observé aucun écart significatif (rapport de cotes (RC) = 0,99; IC de 95 % : 0,93–1,04) dans la solitude autodéclarée en lien avec une augmentation de l’écart interquartile (EI) de l’IVDN (0,06). Cependant, pour le même changement dans la verdure, la probabilité pour les participantes et les participants d’être tout à fait d’accord avec l’énoncé qu’ils « vivent de la solitude dans leur zone locale » était réduite de 15 % (RC = 0,85, IC de 95 % : 0,72–0,99). Et pour chaque augmentation de l’EI de l’IVDN, nous avons observé une baisse de 7 % (RC = 0,93, IC de 95 % : 0,88–0,97) de la prévalence de l’isolement social.

**Conclusion:**

Nos constatations indiquent que la verdure urbaine joue un rôle dans la réduction de la solitude et de l’isolement social chez les citadins et citadines au Canada.

## Introduction

In recent years, loneliness has been increasingly characterized as a global epidemic (Jeste et al. 2020; Jaffe 2023) and some have noted that the worldwide COVID-19 pandemic has exacerbated its impacts (O’Shea et al. 2021; Vlachantoni et al. 2022). Loneliness has been defined as the perceived difference between desired and achieved connection (Peplau and Perlman 1982; Ernst et al. 2022), but can be understood on a social, emotional (Weiss 1973), and existential basis (Bolmsjo et al. 2019). Social loneliness is characterized by a lack of desired interactions with friends and family, whereby emotional loneliness refers to a perceived absence of meaningful relationships with others. A third way of feeling lonely, existential loneliness, involves feeling aimless due to the lack of connection or purpose.

Loneliness and social isolation are often measured concurrently, and while complementary, they are considered distinct constructs (Holt-Lunstad et al. 2015; Cornwell and Waite 2009). Loneliness refers to the subjective rating of feeling alone, whereas social isolation is an objective measure of the degree to which an individual is alone or part of a social network (Courtin and Knapp 2017; de Jong Gierveld and Havens 2004). Specifically, social isolation refers to an individual’s level of contact with their social network which consists of friends, family, community, or workplace members (de Jong Gierveld and van Tilburg 2006). Therefore, it can be possible for someone to feel lonely without being socially isolated, while the corollary that someone can be socially isolated without feeling lonely may hold true.

Loneliness and social isolation are important determinants of health and they have been associated with coronary heart disease and stroke (Valtorta et al. 2018), dementia (Zhou et al. 2018; Fratiglioni et al. 2004), and premature mortality (Perissinotto et al. 2012; Holt-Lunstad et al. 2010). In 2021, an estimated 9% of Canadians aged 65 to 74 and 14% of Canadians aged 75 years and older reported “always” or “often” feeling lonely (Statistics Canada 2021). Similarly, an estimated 14% of Canadians aged 45 to 59 and 10% of Canadians aged 60 and older were characterized as socially isolated (Ramage-Morin 2016). Importantly, the prevalence of both loneliness and social isolation is higher among those with poorer mental health and those who live alone and, in general, tends to increase with increasing age (Ramage-Morin 2016; Evans and Fisher 2022).

The underlying population distribution of Canada is aging rapidly. In 2022, approximately 18.8% of Canadians were 65 years of age and older, and it is anticipated that in 2030 nearly one quarter of Canadians will fall in this age group (Government of Canada 2014). Furthermore, as middle-aged adults transition into older age groups, it is important to consider factors impacting this transition that influence health status. Often this period is marked by changes in employment status and retirement, factors that are commonly associated with changes in social contacts. These shifting demographics underscore the need to develop interventions that prevent or relieve loneliness/social isolation in older adults. Unfortunately, a previous meta-analysis found that interventions tend to be highly individualized, and produce only modest benefits (Masi et al. 2011).

Over the past decade, findings from many studies suggest that features of urban built environments, particularly greenness, improve population health. Access to urban green spaces provides physical and mental health benefits. Studies have linked greenness to improved mental health, and reduced risks of depression (Abraham Cottagiri et al. 2022; Reid et al. 2022), cardiovascular disease (Yu et al. 2023), and mortality (James et al. 2015; Fong et al. 2018; Villeneuve et al. 2012). The possibility that urban greenness reduces the risk of loneliness is supported by findings from recent epidemiological studies (Hammoud et al. 2021; Maas et al. 2009; Astell-Burt et al. 2013, 2021) and the potential for this risk reduction is rooted in collective and relational restoration theory. For example, these theories emphasize the importance of social support between family, friends, community, and pedestrians, opportunities often available in shared green spaces that encourages coming together and promotes social interactions. Through these social interactions, facilitated with access to green spaces, individuals may be provided increased opportunities to achieve greater social connection, thereby replenishing depleted “relational resources” and reducing feelings of loneliness (Hartig 2021). It has also been noted by Astell-Burt that green spaces may “serve as ‘affective sanctuaries’ providing relief from loneliness without requiring connection with other humans” (Astell-Burt et al. 2022a). Specifically, green spaces and connectedness to nature can provide relief from the minutiae of everyday life and serve as a therapeutic setting, giving time for reflection (Butterfield and Martin 2016; Martin et al. 2020). A recent systematic review on the topic by Astell-Burt et al. identified a total of 22 studies of greenness and loneliness, and noted these studies tended to use general measures of loneliness, used a cross-sectional study design, and were undertaken in high-income countries (Astell-Burt et al. 2022a).

The development, support, and implementation of effective urban planning policies designed to improve population health require evidence-based findings. To date, we know of no Canadian community-based study of middle-aged and older adults that has assessed the impacts of urban greenness on loneliness and social isolation. Past work suggests a potential protective effect between urban greenness and depression (Cottagiri et al. 2021), with the largest benefits observed among those with lowest income. This finding suggests urban greening plays an important role in reducing social disparities in health. Recent work has evaluated links between social isolation and social support among participants of the CLSA (Menec et al. 2020), but to date associations between greenness and social isolation in this study population have not been examined. We hypothesized a priori that residential proximity to greenness reduces the risks of both loneliness and social isolation. If so, given that data from the 2016 Canadian Census show that low-income households, visible minorities, and immigrants are more likely to have lower surrounding greenness (Pinault et al. 2021), greening these neighbourhoods holds appeal as an intervention to improve population health and reduce socio-cultural health inequities.

Herein, we report findings from a cross-sectional analysis on loneliness, social isolation, and greenness among participants of CLSA. This study population consists of middle-aged and older adults and is therefore positioned to provide insights on the health benefits of urban built environments as those who are middle-aged transition to older age. Unlike past studies that relied on an overall measure of loneliness, our analysis takes advantage of a survey instrument that asked participants to indicate whether they often feel lonely living in their local area. We hypothesized that associations between greenness and loneliness would be stronger when individuals reported feeling lonely in their local area as compared with an overall measure of loneliness. We also sought to explore whether associations between greenness and loneliness/social isolation were modified by sex or socio-economic status.

## Materials and methods

### Study population and design

We analyzed baseline data from the CLSA survey that collected information from those between the ages of 45 and 85 at the time of enrollment. The CLSA is a prospective cohort study that collected baseline data between 2011 and 2015 with planned follow-up of these participants for approximately two decades. The primary purpose of the CLSA is to advance aging research in Canada by investigating the biological, psychological, and social factors accompanying the aging process from mid-life to older age. We describe some of the key features of the CLSA below; however, the reader is referred to previously published work for a more detailed summary of the study design and methodology (Raina et al. 2009, 2019).

The CLSA consists of a random sample of 51,338 Canadians selected from one of the three sampling frames: (1) Statistics Canada’s Canadian Community Health Survey-Healthy Aging; (2) registries of provincial health care systems; and (3) a random digit dialling of landline telephone. Eligible participants include English- or French-speaking persons able to independently participate physically and cognitively. The survey excludes residents from Canadian territories (Yukon, Nunavut, and Northwest Territories) and Indigenous reserves, institutionalized persons, and full-time Canadian Armed Force members. From the eligible sample, initial recruitment included those who provided contact information (45% participation rate); however, only those who provided consent and completed the required baseline assessments were enrolled (response rate of 10%; Raina et al. 2009, 2019).

The CLSA comprises a Tracking and Comprehensive cohort. The Tracking cohort includes 21,241 participants from the ten Canadian provinces who provided data through telephone interviews. The Comprehensive cohort contains 30,097 participants who provided data through interviews at home and were selected from within 25–50 km of the 11 data collection sites from seven provinces: British Columbia, Alberta, Manitoba, Ontario, Quebec, Nova Scotia, and Newfoundland and Labrador.

**Fig. 1:**
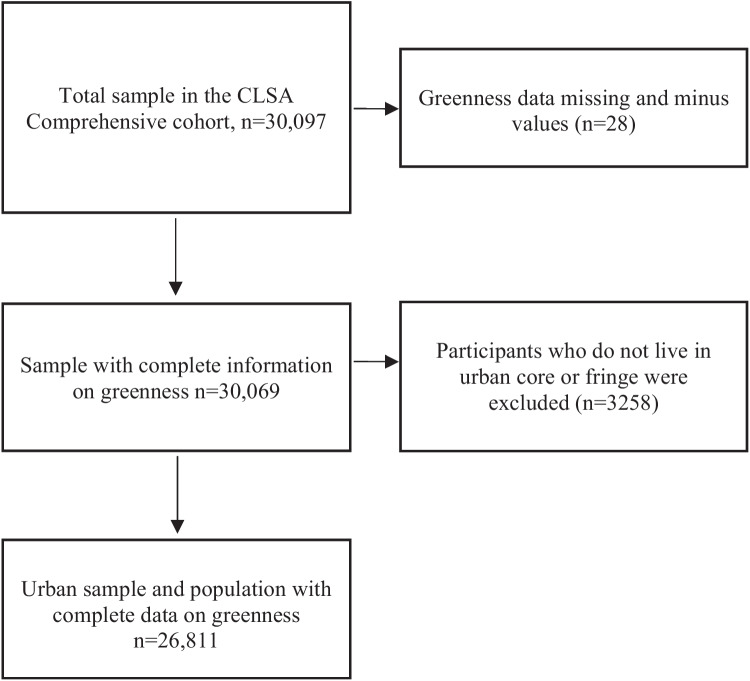
Study flowchart of CLSA participants used to assess association between urban greenness and loneliness

We restricted analyses to the CLSA Comprehensive cohort at baseline as data were collected related to the frequency of interactions participants had with their neighbourhood. This allowed us to discriminate between participants based on their interactions with proximal green space. Further, we restricted analysis to urban participants whose postal codes have relatively high spatial accuracy (typically between 110 and 160 m (Khan et al. 2018)). Compared to urban residences, rural postal codes usually cover much larger land areas, often an entire town or village, and therefore, are not well suited for assigning environmental exposures with a high degree of spatial resolution. Variability in access to and the amount of green space available is a problem specific to urbanites, as rural populations tend to have greater access to green space with little variability throughout the surrounding environment. We classified participants according to whether they resided in urban cores, urban fringes, or urban population centres outside census metropolitan areas (i.e., urban areas with a total population of at least 100,000 people of which 50,000 or more live in the core) and census agglomerations areas (i.e., areas with a core population of at least 10,000 people). The final sample size was 26,811 participants (Fig. 1).

### Loneliness outcome measures

The CLSA survey included two self-reported measures of loneliness. The first measure, defined as “self-reported loneliness,” was found within the condensed version of the Center for Epidemiologic Studies Depression Scale (CES-D-10), which is a ten-item Likert scale questionnaire that assesses depressive symptoms within the last week (Andresen et al. 1994). Specifically, the self-reported measure of loneliness was obtained from the questionnaire item within the CES-D instrument that asked the following: “‘how often did you feel lonely?’ (1 = all of the time (5–7 days), 2 = occasionally (3–4 days), 3 = some of the time (1–2 days), 4 = rarely or never (less than 1–day)).” The second measure of loneliness we modelled was “self-reported loneliness within a neighbourhood”. Participants were asked a series of questions about how they feel about their local area, and this included the question “‘I often feel lonely living in this area’ with possible responses of 1 = strongly agree, 2 = agree, 3 = disagree, 4= strongly disagree”. We modelled both measures of loneliness using a two-level categorization as well as the original four-level classification. The two-level grouping was considered due to the relatively small sample sizes in some cells ($\le$ 1000) combining the first two (yes, lonely) and last two (no, not lonely) answer choices.

### Social isolation outcome measure

The CLSA did not include a direct measure of social isolation. Instead, this measure was derived using a series of 20 questions evaluating participation in social activities and frequency of contact with their social networks (e.g., family, spouses, children, siblings, friends, and neighbours). We defined participants as being socially isolated using the methodology outlined by Menec at al. (2019, 2020). We first created a measure based on the frequency of participation in eight social activities within the past 12 months (i.e., family- or friendship-based, church or religious, sport or physical, educational and cultural, service club and fraternal neighbourhood, community and professional, volunteer or charity work, and recreational activities). Each activity was measured on a scale from 1 to 5 (1 = at least once a day; 2 = at least once a week; 3 = at least once a month; 4 = at least once a year; 5 = never). Second, to measure the frequency of contact with participants, social network questions on marital status; household size; when participants got together with children, siblings, relatives, close friends, and neighbours living outside of their household; and retirement status were included. Finally, a set of continuous variables were used to identify the number of friends, relatives, neighbours, or children included in an individual’s social network (none or $\ge$ 1 person).

### Characterization of surrounding greenness

To characterize the surrounding residential greenness for each participant, we used the Normalized Difference Vegetation Index (NDVI). The NDVI is the most commonly used measure of greenness in epidemiological studies, and provides a sky-based measure of overall ground-based vegetation (Gorelick et al. 2017; Su et al. 2019). We modelled the maximum annual mean value for greenness measured using 30 m resolution images taken by the United States Geological Survey’s Landsat 5 (U.S. Geological Survey 2023a) and Landsat 8 (U.S. Geological Survey 2023b) satellites. The NDVI ranges in values from − 1 to 1, with larger positive values representing denser green vegetation and near-zero values representing bare soil. Negative values indicate the absence of green vegetation, and often represent the presence of water or concrete. Similar to previous studies, we restricted our data to NDVI values between 0 and 1 to isolate green spaces from non-green areas (Crouse et al. 2017; McMorris et al. 2015). NDVI values were aggregated within circular buffers of 250 m, 500 m, and 1 km around each participant’s postal code. We constructed an average NDVI within each buffer and modelled these as our exposure measures. As in previous epidemiological investigations (Cottagiri et al. 2021; McMorris et al. 2015; Su et al. 2019), we focussed on presenting results using a 500 m buffer distance—this corresponds to roughly a 10-min walk. We also conducted sensitivity analyses to explore how our findings changed when other buffer distances were used (250 m and 1000 m). Given that we used baseline data collected between 2011 and 2015, we assigned annual greenness values based on the calendar year that was closest to time of survey completion. Due to the decommissioning of Landsat 5 in 2012, we used the 2013 NDVI surface measures of greenness as provided by Landsat 8. Greenness data could be assigned to all but 28 participants.

### Other covariates

All participants in the CLSA provided information on socio-economic, health behaviour, and lifestyle factors that are relevant to health and aging. Some of these variables were included in our analyses as potential confounders or effect modifiers, including sex (male, female), and age which was categorized as 45–54, 55–64, 65–74, and 75–86 years old. Individuals could only be classified racially as either Caucasian or non-Caucasian due to the small number of individuals from other ethnic backgrounds. Household income was included to assess whether it modified the association between urban greenness and loneliness and social isolation. We also modelled marital status as a risk factor for loneliness and this was a five-level classification variable: (i) single, never married, or never lived with a partner, (ii) married/living with a partner in a common-law relationship, (iii) widowed, (iv) divorced/separated. Stratified analyses were conducted across three income categories (< $50,000, $50,000–$100,000, > $100,000). We adjusted our measures of association (i.e., odds ratios) by province of residence.

We considered several health behaviours that could confound the relationship between greenness and loneliness. The survey assessed alcohol consumption in the following categories: (i) never, (ii) < 1 per month, (iii) 1–3 times a month, (iv) 1–3 times a week, and (v) ≥ 4 times weekly. The survey provided information to construct three categories of smoking: (i) never users, (ii) former smoker, and (iii) current smoker. Participation in physical activity was modelled using a composite score where participants were asked about their frequency of participation in light, moderate, and strenuous activity in the past week (Washburn et al. 1993) and was categorized as follows: (i) no activity (0 days), (ii) little activity (1 to 2 days), (iii) moderate activity (3 to 4 days), and (iv) high activity (5 to 7 days). Other factors assessed include depression and chronic health conditions. Depression was assessed using nine of the ten measures (excluding measure for self-reported loneliness) in the CES-D 10 scale for depression. Finally, yes/no categorizations were used to indicate those with at least one chronic health condition compared to those with none.

Participants were asked how frequently they have been to places in their neighbourhood, which was used to assess neighbourhood interaction in the following categories: (i) daily, (ii) four to six times a week, (iii) one to three times a week, (iv) less than once a week, and (v) no interactions. Categories were regrouped as “4–7 times a week”, “1–3 times a week”, and “no interactions”. Work and retirement status data were also collected and evaluated as potential confounders. Employment was measured using the following categories: (i) employed some of the time (that is, less than 20 h/week), (ii) employed most of the time (that is, less than 30 but more than 20 h/week), (iii) employed all of the time (that is, 30+ h/week), (iv) partly retired, and (v) completely retired. Both categories, working and retirement status, were collapsed and reorganized as “less than 30 hours/week,” “30+ hours/week,” “partly retired,” and “completely retired.” To assess mobility issues, we used participants’ responses to whether they were able to walk (yes/no question). Last, we evaluated household size, grouping homes as either “single person” or “> 1 person”.

### Statistical analysis

First, we calculated descriptive statistics for socio-economic and lifestyle characteristics for study participants. Across these categorical variables, we determined the prevalence of (i) self-reported loneliness and (ii) self-reported loneliness within neighbourhood. We similarly calculated the median NDVI and standard deviation across categories.

To describe the relationship between greenness and loneliness and social isolation, we fit a series of logistic models. The first model was minimally adjusted and included the covariates of age and sex. The second model expanded the first model by also including a series of individual covariates: marital status, household income, mobility, alcohol consumption, and cigarette smoking status. The third model, which represents the fully adjusted model, included the number of neighbourhood interactions, chronic health conditions, household size, depression, and working status.

Stratified analyses were performed to evaluate differences in the strength of the association between greenness and loneliness by sex, household income, neighbourhood interactions, household size, and working status. These analyses were repeated using 250 m and 1000 m buffers of greenness to evaluate the influence of spatial resolution.

Data were analyzed using STATA 16 (StataCorp 2019). Ethics approval for this study was granted by Carleton University (Clearance # 111073).

## Results

Participants were, on average, 63.0 (SD = 10.3) years old, and there were an approximately equal number of men (49.3%) and women (50.7%). Two thirds of participants were married (67.6%), predominantly Caucasian (91.8%), and either partly or completely retired (55.2%). The maximum annual mean NDVI (500 m) was higher for those who were married, had higher household incomes, considered themselves physically active, and lived in multi-person households. Overall, 10.8% of adults perceived being lonely all the time or occasionally, while 6.5% of participants reported that they “strongly agree” or “agree” that they feel lonely living in their local area (Table 1). Female and older participants reported higher prevalence of both measures of loneliness. Conversely, family income was inversely related with loneliness. Specifically, the prevalence of loneliness was 18.5% among those with a household income of less than $50,000, while it was 5.6% among those with incomes ≥ $100,000.

**Table 1:** Descriptive characteristics and self-reported loneliness across socio-economic, health behaviour, and lifestyle variables for urban participants in the comprehensive cohort of the Canadian Longitudinal Study on Aging

Characteristic		Participants		Prevalence of self-reported loneliness^a^	Prevalence of self-reported loneliness in neighbourhood^b^		NDVI^c^	
		*n*	%	(%)	(%)	Median		Std
Sex	Male	13,232	49.3	9.3	5.7	0.769		0.051
	Female	13,579	50.7	12.4	8.1	0.767		0.049
Age	45–54	6718	25.1	8.8	6.9	0.768		0.050
	55–64	8740	32.6	10.7	6.9	0.767		0.050
	65–74	6517	24.3	10.7	6.3	0.767		0.052
	75–86	4836	18.0	14.1	7.5	0.769		0.049
Race	Caucasian	24,407	91.8	10.6	6.6	0.768		0.050
	Non-Caucasian^d^	2180	8.2	13.2	9.4	0.765		0.053
	Missing	224						
Marital status	Single, never married	2451	9.1	17.6	12.6	0.756		0.057
	Married/common law	18,129	67.6	6.4	4.6	0.770		0.049
	Widowed	2557	9.5	26.2	11.5	0.767		0.050
	Divorced/separated	3666	13.7	17.8	11.3	0.763		0.053
	Missing	8						
Household income	< $50,000	6983	27.9	18.5	11.3	0.762		0.053
	$50,000–$100,000	8802	35.1	9.7	5.7	0.768		0.050
	> $100,000	9260	37.0	5.6	4.5	0.772		0.048
	Missing	1766						
Employment	< 30 h/week	1793	6.7	10.5	7.9	0.767		0.051
	30+ h/week	8841	33.0	8.1	5.6	0.769		0.050
	Partly retired	2891	10.8	10.8	6.1	0.766		0.052
	Completely retired	11,906	44.4	12.0	7.1	0.769		0.050
	Missing	1380						
Province	Alberta	2819	10.5	10.7	5.7	0.728		0.057
	British Columbia	5731	21.4	9.6	6.8	0.780		0.050
	Manitoba	3031	11.3	11.1	5.4	0.733		0.041
	Newfoundland and Labrador	1998	7.5	10.1	5.3	0.798		0.029
	Nova Scotia	2696	10.1	9.4	4.9	0.766		0.031
	Ontario	5668	21.1	10.1	5.8	0.783		0.030
	Quebec	4868	18.2	14.2	11.5	0.759		0.058
Alcohol consumption	Never	3122	11.9	15.8	8.9	0.767		0.052
	< 1 per month	3288	12.6	13.8	9.5	0.764		0.050
	1–3 times a month	4477	17.1	10.7	7.0	0.766		0.049
	1–3 times a week	8364	32.0	9.1	5.9	0.769		0.051
	≥ 4 times weekly	6915	26.4	9.1	5.7	0.770		0.050
	Missing	645						
Smoking status	Never	12,782	47.7	9.8	6.4	0.769		0.049
	Former	11,631	43.4	10.9	6.8	0.768		0.051
	Current	2397	8.9	16.2	9.9	0.762		0.055
	Missing	1						
Physical activity	High activity	1003	3.9	8.3	3.9	0.772		0.050
	Moderate activity	6308	24.6	8.3	5.8	0.769		0.049
	Little activity	16,233	63.3	11.1	7.1	0.767		0.051
	No activity	2102	8.2	14.3	10.0	0.767		0.048
	Missing	1165						
Chronic health conditions	No	24,651	91.9	11.3	7.1	0.768		0.050
	Yes	1975	7.4	5.7	4.7	0.767		0.053
	Missing	185						
Neighbourhood interactions	No interactions	552	2.1	16.9	13.43	0.767		0.047
	1–3 times	2283	8.5	16.0	11.01	0.764		0.049
	4–7 times	23,965	89.4	10.2	6.35	0.768		0.050
	Missing	11						
Mobility issues	No	26,249	97.9	10.7	6.7	0.768		0.050
	Yes	560	2.1	20.3	15.2	0.766		0.053
	Missing	2						
Household size	Single person	6287	23.5	21.6	12.6	0.761		0.054
	> 1 person	20,524	76.6	7.6	5.2	0.770		0.049
All participants		26,811	100.0	10.8	6.5	0.768		0.050

^a^Participants whose item response was either “All of the time (5–7 days)” or “Occasionally (3–4 days)” were classified as lonely

^b^Participants whose item response was either “Strongly Agree” or “Agree” were classified as lonely

^c^The maximum annual mean NDVI within a 500 m circular buffer from the centroid of participant’s postal code

^d^Non-white: Black (207), Korean/Filipino/Japanese/Chinese (305), Southeast/South Asian (323), Arab/West Asian (120), Other + Multiple (257), Indigenous (1127)

**Fig. 2:**
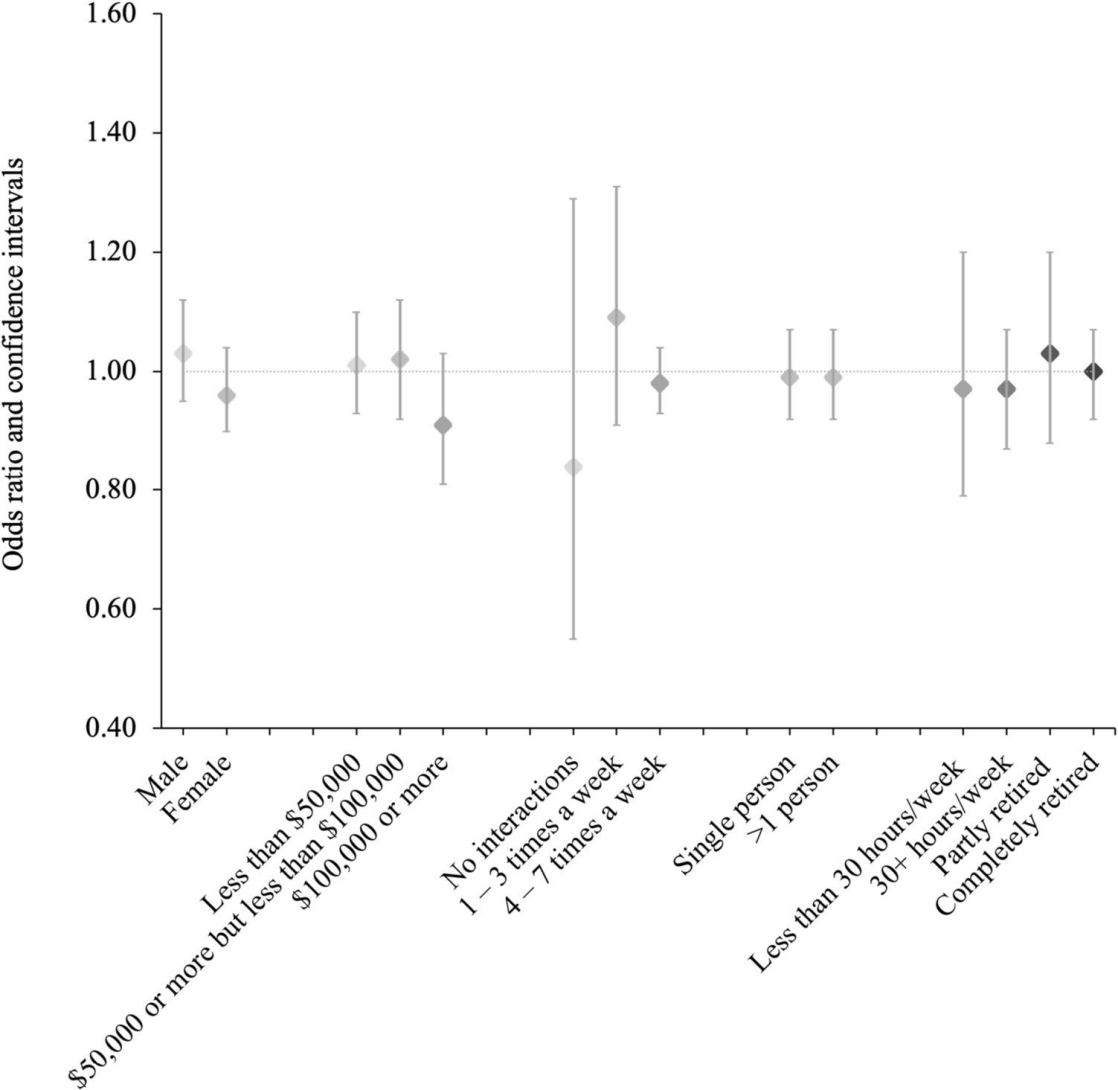
Odds ratios (95% CI) of self-reported loneliness for an interquartile range increase in urban greenness

In unadjusted models, we found an inverse association between residential greenness (500 m) and self-reported loneliness (see Table 2). However, following adjustment for other covariates, the association was attenuated and no longer statistically significant. The odds ratio (OR) of being lonely in relation to an interquartile range increase in the NDVI was 0.99 (95% CI 0.93–1.04). Similarly, no association between greenness and loneliness was observed when a four-level classification of loneliness was used (see Table 2). We did not identify any statistically significant associations between greenness and self-reported loneliness in stratified analyses across sex, income, neighbourhood interactions, working status, or household size (see Fig. 2).

**Table 2:** Adjusted odds ratios (ORs) for an interquartile range increase in greenness (based on a 500 m buffer) for selected loneliness measures and social isolation among urban participants of the Canadian Longitudinal Study on Aging (*n* = 26,811)

Loneliness		Participants	Model 1		Model 2		Model 3	
			OR	95% CI	OR	95% CI	OR	95% CI
Self-reported loneliness ^a^	No	23,808	1.00		1.00		1.00	
	Yes	2896	0.91	0.87–0.95	0.98	0.93–1.03	0.99	0.93–1.04
	Rarely or never	19,660	1.00		1.00		1.00	
	Some of the time	4148	0.89	0.86–0.93	0.95	0.91–0.99	0.97	0.93–1.01
	Occasionally	2325	0.89	0.86–0.93	0.96	0.91–1.01	0.98	0.93–1.04
	All of the time	571	0.89	0.81–0.97	1.01	0.91–1.11	0.99	0.87–1.11
Self-reported loneliness	No	23,736	1.00		1.0		1.0	
within neighbourhood ^b^	Yes	1754	0.88	0.83–0.93	0.93	0.88–0.98	0.94	0.88–0.99
	Strongly disagree	10,201	1.00		1.0		1.0	
	Disagree	13,535	0.91	0.88–0.94	0.94	0.91–0.97	0.95	0.92–0.98
	Agree	1519	0.84	0.79–0.89	0.90	0.85–0.96	0.91	0.85–0.98
	Strongly agree	235	0.82	0.71–0.94	0.86	0.74–0.99	0.85	0.72–0.99
Social isolation	No	22,468	1.0		1.0		1.0	
	Yes	4343	0.82	0.79–0.85	0.91	0.87–0.95	0.93	0.88–0.97

^a^Participants whose item response was either “All of the time (5–7 days)” or “Occasionally (3–4 days)” were classified as lonely

^b^Participants whose item response was either “Strongly Agree” or “Agree” were classified as lonely

Model 1: Adjusted for age and sex

Model 2: Adjusted for age, sex, race, marital status, household income, alcohol consumption, smoking status, and mobility issues

Model 3: Adjusted for age, sex, race, marital status, household income, alcohol consumption, smoking status, mobility issues, chronic health conditions, neighbourhood interactions, household size, depression, and working status

In contrast, we found stronger protective effects of greenness for neighbourhood-based measures of loneliness. An interquartile range increase in the NDVI was associated with a reduced odds of self-reported loneliness within one’s neighbourhood (OR = 0.94, 95% CI 0.88–0.99). An exposure-response pattern was evident when a four-level classification of neighbourhood loneliness was used. An interquartile range increase in greenness was associated with reduced odds of participants indicating that they “agreed” or “strongly agreed” the neighbourhood contributed to their loneliness (OR = 0.91, 95% CI 0.85–0.98; OR=0.85, 95% CI 0.72–0.99) relative to those who indicated they “disagreed” or “strongly disagreed.” Similarly, in the fully adjusted model, greenness was associated with a 7% (OR = 0.93, 95% CI 0.88–0.97) reduced risk of being socially isolated.

**Fig. 3:**
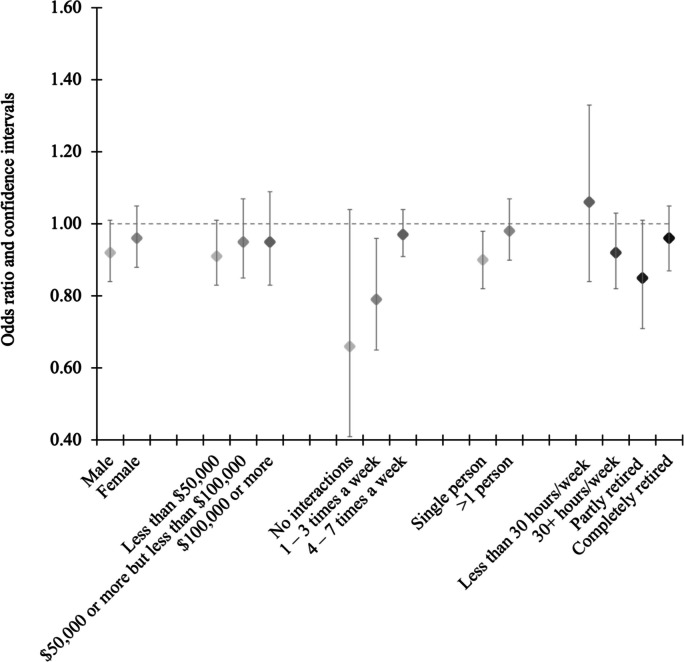
Odds ratios (95% CI) of self-reported loneliness within neighbourhood for an interquartile range increase in urban greenness among urban participants of the Canadian Longitudinal Study of Aging (*n* = 26,811)

Stratified analyses found inverse associations between greenness and the neighbourhood-based measure of loneliness were strongest among those who had between one and three neighbourhood interactions a week (Fig. 3). We also observed that increased greenness was associated with lower odds of reporting feeling lonely in an area among men and among those who lived alone.

Findings from our sensitivity analyses comparing the strength of association between greenness and loneliness across different buffer intervals are presented in Supplementary Table S1. For the neighbourhood-based measure of loneliness, the inverse association with greenness was strongest when a 500 m buffer was modelled. We found no association between our overall measure of loneliness and greenness for any of the three buffer distances that we modelled.

## Discussion

Our analyses of CLSA baseline cross-sectional data suggest that urban greenness reduces the prevalence of loneliness and social isolation. These findings coupled with earlier work on depression in the same study population (Abraham Cottagiri et al. 2022) provide evidence that greening likely represents an important strategy to improve the mental health of Canadians.

While we found that overall vegetation produced a small decrease in odds of neighbourhood-based loneliness, more work is needed to better understand what types of greenness contribute most to this relationship. Recent findings from a prospective cohort study in Australia found stronger association between tree canopy and loneliness relative to other forms of vegetation (Astell-Burt et al. 2022b). Among women, a 10% increase in total green space was associated with reduced odds of prevalent social loneliness (OR = 0.95, 95% CI 0.91–0.95), while a 10% increase in tree canopy was associated with an 11% (OR = 0.89, 95% CI 0.85–0.92) reduction in the odds of loneliness. Similar patterns were evident among men. In contrast, this Australian study found that an increase in open grass was associated with increased risks of loneliness. While our use of the NDVI facilitates comparisons with previous Canadian work, for us, it is becoming increasingly evident that there is an important need to evaluate other measures of greenness, and measures such as tree canopy and the Green View Index (Villeneuve et al. 2018) should be the focus of future Canadian epidemiological studies.

We also recognize that many of the larger Canadian health survey datasets are non-representative of the Canadian population. The CLSA participants were predominantly Caucasian, and therefore, we should be cautious to generalize these findings to all Canadian adults. A similar concern applies to the findings from McMorris et al. in their study involving participants of the Canadian Community Health Survey (CCHS) (McMorris et al. 2015). More research is needed to assess the possible benefits of the urban built environments in more diverse study populations. Large survey populations in Canada tend to over-represent individuals from more affluent, educated, and less ethnically diverse groups (Brayne and Moffitt 2022; Norberg et al. 2021). An alternative survey dataset such as the Canadian Census Cohort (CanCHEC) (Crouse et al. 2017) assembled from the national census is a representative sample of Canadians; however, identifying mental health outcomes such as depression and loneliness is problematic as these questions are not asked on the census, and not readily obtained through record linkage to other administrative health datasets. The CanCHEC cohorts, because they are census-based, also lack important data on other possible risk factors.

We found that the protective effects of greenness and the neighbourhood-based measure of greenness were strongest when the 500 m buffer was used (as compared to 250 m and 1 km buffers). The benefits of greening on loneliness in Australia were based on greenness measures within 1.6 km distance from participants’ homes (Astell-Burt et al. 2022b). Elsewhere, a study in the United Kingdom that included approximately 200,000 participants, that also modelled residential greenness using a 500 m buffer, found a statistically significant reduced risk of social isolation (OR = 0.97; 95% CI 0.95‒0.99) (Lai et al. 2021). Most studies of greenness and loneliness have not evaluated differential effects across different buffers; however, such analyses can be helpful to identify relevant pathways whereby greenness can impact health. In addition, identifying which buffer distances produce the greatest benefits can help inform the design of greening interventions.

We undertook a cross-sectional analysis of the CCHS. As noted by Astell-Burt et al., this is similar to most other studies on this topic (Astell-Burt et al. 2022a). The use of the cross-sectional study design is limited for evaluating causal associations. Moreover, cross-sectional data analysis typically carries with it a limited ability to undertake mediation analysis (Cain et al. 2018) for behaviours such as physical activities and social interactions that may ultimately be the pathways whereby greenness may reduce loneliness and social isolation. Longitudinal analyses also have advantages for assessing self-selection bias related to place of residence and greenness. Namely, those of better health and better income may be more likely to choose to live in greener areas, rather than greenness itself conferring the benefit (Gailey 2022; McCormack 2017). For all these reasons, efforts to apply longitudinal analyses of greenness and health should be prioritized over cross-sectional data.

## Conclusion

Our findings add to the growing literature that urban greenness reduces loneliness and social isolation. Continued follow-up of this cohort would help to better understand how some features of urban built environments may have helped mitigate loneliness during the COVID-19 pandemic. Regardless, loneliness and social isolation continue to represent important public health concerns that impact individuals of all ages, but particularly older adults. The US Surgeon General recently declared that loneliness is a public health crisis with impacts on mortality the equivalent of smoking up to 15 cigarettes a day (Jaffe 2023). For these reasons, and given the many other ways that greenness has been shown to improve health (James et al. 2015), the future design of urban areas should move quickly to implement greening initiatives.

## Contributions to knowledge

What does this study add to existing knowledge?

- This study provides insight on how features of urban built environments, specifically greenness, are related to loneliness and social isolation in older adults.
- The study found that residential proximity to green spaces reduced the prevalence of being lonely within the neighbourhood, and social isolation. These associations were stronger for those in single-person households.

What are the key implications for public health interventions, practice, or policy?

- These findings provide further support for implementing greening interventions in urban areas to improve population health.

### Supplementary Information

Below is the link to the electronic supplementary material.

Supplementary file1 (DOCX 51 KB)

## Data Availability

Access to data by others is not allowed due to informed consent and ethics in place.

## Code availability

NA
