# Supplementary material for: Does urban greenness reduce loneliness and social isolation among Canadians? A cross-sectional study of middle-aged and older adults of the Canadian Longitudinal Study on Aging (CLSA)
Source: Can J Public Health. 2023 Dec 29;115(2):282–95. doi: 10.17269/s41997-023-00841-x (PMC11006650; doi:10.17269/s41997-023-00841-x)
Supplement: Supplementary file 1 — Supplementary file1 (DOCX 51 KB) [file 41997_2023_841_MOESM1_ESM.docx]

Table S1: Adjusted odds ratios (ORs) for an interquartile range (IQR) increase in greenness for different buffers of loneliness measures among urban participants of the Canadian Longitudinal Study on Aging (n=26,811)

| Loneliness |  | Participants |  | 250 m Buffer | |  | 500 m Buffer | |  | 1000 m Buffer | |
| --- | --- | --- | --- | --- | --- | --- | --- | --- | --- | --- | --- |
|  |  |  |  | Odds Ratio | 95% CI |  | Odds Ratio | 95% CI |  | Odds Ratio | 95% CI |
| Perceived loneliness ^a^ | No | 23,808 |  | 1.0 |  |  | 1.0 |  |  | 1.0 |  |
|  | Yes | 2896 |  | 1.00 | 0.95 – 1.06 |  | 0.99 | 0.93 – 1.04 |  | 0.96 | 0.91 – 1.02 |
|  |  |  |  |  |  |  |  |  |  |  |  |
|  | Rarely or never | 19,660 |  | 1.0 |  |  | 1.0 |  |  | 1.0 |  |
|  | Some of the time | 4,148 |  | 0.98 | 0.94 – 1.02 |  | 0.97 | 0.93 – 1.01 |  | 0.97 | 0.93 – 1.02 |
|  | Occasionally | 2,325 |  | 0.99 | 0.94 – 1.05 |  | 0.98 | 0.93 – 1.04 |  | 0.96 | 0.90 – 1.02 |
|  | All of the time | 571 |  | 1.03 | 0.91 – 1.16 |  | 0.99 | 0.87 – 1.11 |  | 0.95 | 0.84 – 1.08 |
|  |  |  |  |  |  |  |  |  |  |  |  |
| Perceived loneliness | No | 23,736 |  | 1.0 |  |  | 1.0 |  |  | 1.0 |  |
| within neighbourhood ^b^ | Yes | 1754 |  | 0.96 | 0.90 – 1.02 |  | 0.94 | 0.88 – 0.99 |  | 0.96 | 0.90 – 1.02 |
|  |  |  |  |  |  |  |  |  |  |  |  |
|  | Strongly Disagree | 10,201 |  | 1.0 |  |  | 1.0 |  |  | 1.0 |  |
|  | Disagree | 13,535 |  | 0.94 | 0.90 – 0.97 |  | 0.95 | 0.92 – 0.98 |  | 0.92 | 0.88 – 0.95 |
|  | Agree | 1,519 |  | 0.92 | 0.86 – 0.98 |  | 0.91 | 0.85 – 0.98 |  | 0.97 | 0.90 – 1.05 |
|  | Strongly Agree | 235 |  | 0.90 | 0.77 – 1.06 |  | 0.85 | 0.72 – 0.99 |  | 0.95 | 0.79 – 1.15 |
|  |  |  |  |  |  |  |  |  |  |  |  |
| Social isolation | No | 22,468 |  | 1.0 |  |  | 1.0 |  |  | 1.0 |  |
|  | Yes | 4,343 |  | 0.93 | 0.89 – 0.97 |  | 0.93 | 0.88 – 0.97 |  | 0.98 | 0.93 – 1.03 |

^a^ Participants whose item response was either ‘All of the time (5-7 days)’ or ‘Occasionally (3-4 days)’ were classified as lonely.

^b^ Participants whose item response was either ‘Strongly Agree’ or ‘Agree’ were classified as lonely.

*Odds ratios adjusted for age, sex, race, marital status, household income, alcohol consumption, smoking status, mobility issues, chronic health conditions, neighbourhood interactions, household size, depression, and working status.

**Table S2:** Adjusted odds ratios (ORs) for an interquartile range increase in greenness (based on a 500 m buffer) for selected loneliness measures among urban participants of the Canadian Longitudinal Study of Aging for employment status

|  | Adjusted Odds Ratios (OR) for loneliness  *(Those who are not lonely are the reference group)* | | | | | | | |
| --- | --- | --- | --- | --- | --- | --- | --- | --- |
| Loneliness | Completely retired ^c^ | | Partly retired ^c^ | | 30+ hours/week ^c^ | | Less than 30 hours/week ^c^ | |
|  | (n= 11,906) | | (n= 2891) | | (n= 8841) | | (n= 1793) | |
|  | OR | 95% CI | OR | 95% CI | OR | 95% CI | OR | 95% CI |
| Self-reported loneliness ^a^ |  | | | |  |  |  | |
| No | 1.0 |  | 1.0 |  | 1.0 |  | 1.0 |  |
| Yes ^b^ | 1.0 | 0.92 – 1.07 | 1.03 | 0.88 – 1.20 | 0.97 | 0.87 – 1.07 | 0.97 | 0.79 – 1.20 |
|  |  |  |  |  |  |  |  |  |
| Self-reported loneliness |  | | | |  | |  | |
| within neighbourhood ^b^ |  |  |  |  |  |  |  |  |
| No | 1.0 |  | 1.0 |  | 1.0 |  | 1.0 |  |
| Yes ^b^ | 0.96 | 0.87 – 1.05 | 0.85 | 0.71 – 1.01 | 0.91 | 0.82 – 1.03 | 1.06 | 0.84 – 1.33 |
|  |  |  |  |  |  |  |  |  |
| Social isolation |  |  |  |  |  |  |  |  |
| No | 1.00 |  | 1.00 |  | 1.00 |  | 1.00 |  |
| Yes | 0.87 | 0.82 – 0.93 | 0.89 | 0.78 – 1.02 | 0.85 | 0.78 – 0.93 | 0.89 | 0.73 – 1.08 |

^a^ Participants whose item response was either ‘All of the time (5–7 days)’ or ‘Occasionally (3–4 days)’ were classified as lonely.

^b^ Participants whose item response was either ‘Strongly Agree’ or ‘Agree’ were classified as lonely.

^c^ Adjusted for age, sex, race, marital status, household income, alcohol consumption, smoking status, mobility issues, chronic health conditions, neighbourhood interactions, household size, and depression.

**Table S3:** Adjusted odds ratios (ORs) for an interquartile range increase in greenness (based on a 500 m buffer) for selected loneliness measures among urban participants of the Canadian Longitudinal Study of Aging for those with mobility issues

|  | Adjusted Odds Ratios (OR) for loneliness outcomes  *(Those who are not lonely are the reference group)* | | | |
| --- | --- | --- | --- | --- |
| Loneliness | No mobility issues ^c^ | | Yes, mobility issues ^c^ | |
|  | (n=26,249) | | (n=560) | |
|  | OR | 95% CI | OR | 95% CI |
| Self-reported loneliness ^a^ |  | | | |
| No | 1.0 |  | 1.0 |  |
| Yes ^b^ | 0.99 | 0.94 – 1.04 | 0.96 | 0.68 – 1.36 |
|  |  |  |  |  |
| Self-reported loneliness |  | | | |
| within neighbourhood ^b^ |  |  |  |  |
| No | 1.0 |  | 1.0 |  |
| Yes ^b^ | 0.95 | 0.89– 1.01 | 0.77 | 0.51 – 1.16 |
|  |  |  |  |  |
| Social isolation |  |  |  |  |
| No | 1.0 |  | 1.0 |  |
| Yes | 0.92 | 0.89 – 0.97 | 0.80 | 0.58 – 1.11 |

^a^ Participants whose item response was either ‘All of the time (5-7 days)’ or ‘Occasionally (3-4 days)’ were classified as lonely.

^b^ Participants whose item response was either ‘Strongly Agree’ or ‘Agree’ were classified as lonely.

^c^ Adjusted for age, sex, race, marital status, household income, alcohol consumption, smoking status, chronic health conditions, neighbourhood interactions, household size, depression, and working status.
